# Research on the impact of basic psychological needs satisfaction on career adaptability of Chinese college students

**DOI:** 10.3389/fpsyg.2023.1275582

**Published:** 2023-10-17

**Authors:** Min Xu, Haidong Lu, Qi Guo

**Affiliations:** ^1^School of Psychology, Northeast Normal University, Changchun, China; ^2^School of Psychological and Educational Sciences, Zaozhuang University, Zaozhuang, China

**Keywords:** basic psychological needs satisfaction, career adaptability, career decision self-efficacy, career outcome expectations, Chinese college students

## Abstract

**Introduction:**

With social changes, the realization of smooth and satisfactory employment of college students is an issue that requires deep thought. Basic psychological needs satisfaction play an important role as a guiding factor that affects students’ career adaptability. This study aims to explore the relationship and mechanism between the satisfaction of students’ basic psychological needs satisfaction and their career adaptability.

**Methods:**

A survey is conducted among students from six different universities across the country, using the Basic Psychological Needs Satisfaction Scale, the Career Adaptability Scale, the Career Decision Self-Efficacy Scale, and the Career Outcome Expectations Scale.

**Results:**

The satisfaction of basic psychological needs, career decision self-efficacy, and career outcome expectations are all significantly positively correlated with career adaptability.Career decision self-efficacy plays a partial mediating role between basic psychological needs satisfaction and career adaptability among college students. The moderated mediation model found that career outcome expectations play a regulatory role in the effect of career decision self-efficacy on career adaptability.

**Discussion:**

College students’ basic psychological need satisfaction can positively predict career adaptability directly and indirectly affect career adaptability through career decision self-efficacy, and career outcome expectations have a moderating role between career decision self-efficacy and career adaptability.

## 1. Introduction

The scale and increment of college graduates have reached historical highs, with 11.58 million students graduating in 2023. In response, employers increasingly prefer employees who demonstrate flexibility and adaptability (Klehe et al., 2011), as the employment goals of college students have transitioned from seeking traditional secure jobs to personalized and diverse career paths (Ma and Feng, 2021). Career adaptability, defined as the ability to self-adjust in response to task or environmental changes during different career development stages (Savickas, 1997; Savickas and Porfeli, 2012), is recognized as a crucial factor in career development and success (Guan and Li, 2015; Ma and Feng, 2021). Enhancing college students’ career adaptability is imperative to address the challenge of graduate employ ability, making it essential to explore the factors and mechanisms influencing their career adaptability.

Career adaptability initially originated from Super’s career development theory in the discussion of career maturity. During the transition from school to work and from one job to another, individuals need to constantly adapt to environmental changes, facing various adaptability challenges rather than mature tasks (Savickas, 1997). The concept of career adaptability has evolved through multiple stages. It was first introduced as a concept of career maturity in Career Development Theory of Super and Knasel (1981), and Savickas (1997) defined it as an individual’s ability to self-regulate to cope with predictable career tasks and to face unpredictable career changes or problems. In 2005, career adaptability became part of construction career theory (CCT) and was defined as psycho-social constructs, in which individuals are prepared and provided resources for dealing with current and upcoming career development tasks, transitions, crises, or unexpected events (Savickas, 2005). Later, Savickas and Porfeli (2012) defined it as a type of social psychological resource or psychological capital. Career adaptability is the ability of individuals to adapt to environmental changes, cope with career issues, transitions, and crises, serving as a special form of human resource and a type of social psychological resource (Savickas and Porfeli, 2012).

The influencing factors of career adaptability are mainly concentrated at the individual level, with relatively fewer factors involving external environmental aspects. Research at the individual level focuses on proactive personality traits, the big five personality, and positive psychological qualities. For example, many studies have consistently found that proactive personality traits can positively predict the development of career adaptability (Tolentino et al., 2014; Yang et al., 2021). Additionally, hope and optimism can promote the formation and development of individual career adaptability (Tolentino et al., 2014; Wilkins et al., 2014; Negru-Subtirica and Pop, 2016; Santilli et al., 2017). Research on external environmental factors is relatively limited and mainly focuses on objective family factors, family styles, and social support. For example, the higher the family’s socioeconomic status, the higher the career adaptability level of college students, while those in the middle range may perform worse (Zhao, 2012). Moreover, school support can also predict career adaptability (Hirschi, 2009). Therefore, social support systems and the environment have a certain impact on the development of career adaptability. For college students, whether basic psychological needs affect the development of career adaptability and how they impact career adaptability development still require further research and exploration. This study aims to explore the positive predictive role of basic psychological needs satisfaction on career adaptability among college students and investigate the mechanisms underlying this relationship.

Deci and Ryan proposed Self-Determination Theory, of which the most central theory is Basic Psychological Need Theory. It elaborates the process of internal motivation and internalization of external motivation promoted by the external environment from an organic dialectical perspective, and is a theory of motivation for human behavior. Basic psychological need theory believes that human beings have autonomy needs, competence needs, and relationship needs, and when the three psychological needs are satisfied, it will promote internal motivation, which is conducive to the positive development and behavior of the individual (Deci and Ryan, 2000). If any of the needs are not satisfied or frustrated, it will affect the growth and development of the individual (Wu et al., 2018). According to the theory, basic psychological needs fulfillment can promote individual development, but most of the research focuses on life satisfaction and happiness, etc., and there are fewer studies on individual career development. For example, basic psychological need fulfillment of college students promotes happiness (Yu et al., 2022).

Currently, there are relatively few studies on the relationship between the satisfaction of basic psychological needs and career development, and even fewer studies on career adaptability. Additionally, most of the existing research is conducted in foreign countries, with very limited research on this topic available in domestic literature. It has demonstrated that the satisfaction of basic psychological needs promotes autonomy and skill variety in career work (Van den Broeck et al., 2016), enhances career engagement (Toyama et al., 2022), and career satisfaction (Busque-Carrier et al., 2022; Yang et al., 2022),while reducing career burnout (Van den Broeck et al., 2008), career pressure, work–family conflict, and turnover (Van den Broeck et al., 2016). Prior studies have shown a significant positive relationship between basic psychological needs satisfaction and career adaptability (Gulsen and Sahin, 2022)，and the satisfaction of basic psychological needs promotes career adaptability. However, the predictive power of basic psychological needs satisfaction for career adaptability and the underlying mechanisms remains under-explored, necessitating further empirical research. Therefore, we assume that as:

*Hypothesis 1*: The satisfaction of basic psychological needs positively predicts the development of career adaptability in college students.

Currently, there is no research on the relationship between basic psychological needs, career decision self-efficacy, and career adaptability. However, there are studies related to the associations between basic psychological needs and career decision self-efficacy, basic psychological needs and career adaptability, and career decision self-efficacy and career adaptability. Moreover, according to Social Cognitive Career Theory (SCCT), individual’s career behavior is influenced by cognitive factors, and it states that career decision self-efficacy and career outcome expectations are core cognitive factors (Lent et al., 1994). Therefore, introducing career decision self-efficacy and career outcome expectations as variables can further explore the mechanism of how the satisfaction of basic psychological needs affects career adaptability in college students.

Career decision self-efficacy refers to an individual’s confidence in their ability to successfully accomplish career-related tasks during the decision process (Betz and Hackett, 1981). Career decision self-efficacy is a cognitive factor in career development that is more effective in predicting career choice behaviors compared to individual interests, values, and abilities. It is considered a key factor in achieving career goals (Lent et al., 1994, 2003). It has been shown that when basic psychological needs are satisfied, teaching self-efficacy can be enhanced (Cai et al., 2018), leading to higher career satisfaction. On the other hand, if basic psychological needs are thwarted or unmet, it results in lower levels of career satisfaction (Busque-Carrier et al., 2022). Moreover, career decision self-efficacy is found to promote career maturity (Ye et al., 2020) and significantly positively correlated with career adaptability (Zhang et al., 2022). It also directly predicts individuals’ career adaptability levels (Li et al., 2021). Based on the findings mentioned above, it can be concluded that basic psychological needs satisfaction can influence career adaptability through the mediating role of career decision self-efficacy among college students. In other words, career decision self-efficacy acts as an intermediary variable between basic psychological needs satisfaction and career adaptability. Therefore, we assume that as:

*Hypothesis 2*: Career decision self-efficacy mediates the relationship between basic psychological needs satisfaction and career adaptability.

Career outcome expectations refer to an individual’s belief about the results of specific career decisions (Betz and Voyten, 1997), representing estimations of the consequences of career-related behaviors. Higher career outcome expectations have been shown to motivate positive work attitudes (Hawley McWhirter et al., 2000), and they are significantly positively correlated with career decision self-efficacy (Betz and Voyten, 1997; Chen, 2019). Also, they not only exhibit a significant positive correlation with career adaptability but also positively predicts career adaptability (Chen, 2019). According to social cognitive career theory, career outcome expectations combine with career decision self-efficacy to influence career behaviors, constituting vital cognitive factors in career development (Lent et al., 1999). Additional research has shown that career outcome expectations moderate the influence of career decision self-efficacy on career maturity (Fu, 2015) and career development attitudes (Shi, 2013). Based on the comprehensive findings, career outcome expectations, emerging as a crucial cognitive factor influencing career behaviors, are significantly correlated with both career decision self-efficacy and career adaptability. Therefore, we assume that as:

*Hypothesis 3*: Career outcome expectations moderate the direct impact of career decision self-efficacy on career adaptability.

**Figure 1:**
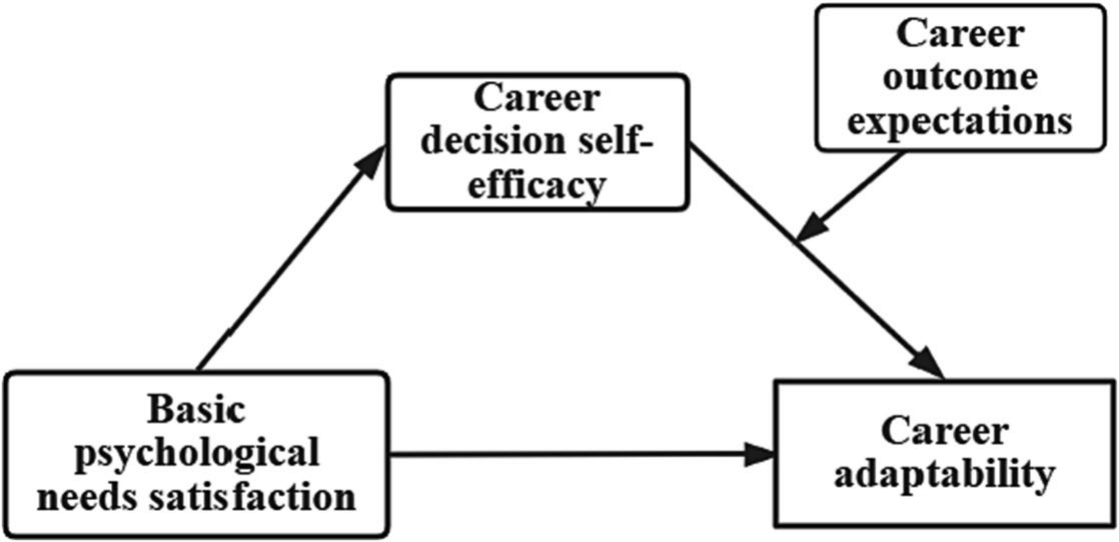
Moderated mediation model.

In summary, this study aims to conduct an in-depth investigation into the relationship and underlying mechanisms between basic psychological needs satisfaction and career adaptability using a moderated mediation model. The specific objectives of this research are (1) to explore the influence of basic psychological needs satisfaction on career adaptability among college students and whether career decision self-efficacy mediates this relationship, and (2) to examine whether career outcome expectations moderate the latter half of the mediating process, i.e., whether career outcome expectations moderate the influence of career decision self-efficacy on career adaptability (see Figure 1).

## 2. Materials and methods

### 2.1. Participants

The present study extracted a total of six universities’ undergraduate students in Guangdong, Jiangsu, Shandong, and Liaoning provinces in China. The survey was conducted using the online platform Wenjuanxing, and a total of 782 questionnaires were collected. After removing 92 invalid questionnaires, there were 690 valid questionnaires (88.23%). Among the respondents, there were 166 male students (24.1%) and 524 female students (75.9%). In terms of academic years, there were 318 freshmen (46.1%), 316 sophomores (45.8%), 24 juniors (3.5%), and 32 seniors (4.6%). Additionally, 457 students (66.2%) came from rural areas, while 233 students (33.8%) came from urban areas. 278 (40.3%) students majored in Arts and History, 236 (34.2%) students majored in Science and Engineering, and 176 (25.5%) students majored in Arts and Sciences.

### 2.2. Measures

#### 2.2.1. Basic psychological needs satisfaction

A Chinese version of the basic psychological need satisfaction scale (Sheldon and Niemiec, 2006), translated and revised by Du Jian was used in this study (Du et al., 2020). The scale assessed three dimensions of basic psychological needs: autonomy, competence, and relatedness, with a total of nine items, consisting of three items for each dimension. Participants rated each item on a seven-point scale, where higher scores indicate a higher level of satisfaction with their basic psychological needs. The overall internal consistency reliability of the scale in this study was reported to be 0.940.

#### 2.2.2. Career decision self-efficacy

A Chinese version of Betz and Taylor’s career decision self-efficacy scale was used in this study (Peng and Long, 2001). The scale included 39 items organized into five dimensions: self-appraisal (six items), information gathering (nine items), goal selection (nine items), plan formulation (eight items), and problem-solving (seven items). Participants rated each item on a five-point scale, where higher scores indicate a higher level of self-confidence in their career decision abilities. The overall internal consistency reliability of the scale in this study was reported to be 0.976.

#### 2.2.3. Career outcome expectations

A Chinese version of the career outcome expectations-revised scale, developed by Hawley McWhirter et al. (2000) and translated and revised for college students by Fu Yuhong was used in this study (Fu, 2015). The scale was a uni-dimensional scale comprising 12 items. Participants rated each item on a four-point scale, ranging from strongly disagree to strongly agree. Higher scores on the scale indicated more positive career outcome expectations. The overall internal consistency reliability of the scale in this study was reported to be 0.911.

#### 2.2.4. Career adaptability

A Chinese version of the career adaptability scale (Savickas and Porfeli, 2012), revised by Hou Zhijin, was used in this study. The scale measured career adaptability in college students across four dimensions: career concern, career control, career curiosity, and career confidence. It consisted of 24 items, with each dimension comprising six items. Participants rated each item on a five-point scale, where higher scores indicate stronger career adaptability (Hou et al., 2012). The overall internal consistency reliability of the scale in this study was reported to be 0.962.

### 2.3. Data analyses

Common method bias test and descriptive statistical analysis were performed by SPSS 22.0, and mediating and moderating effects testing were conducted with the PROCESS plugin in SPSS 22.0.

## 3. Results

### 3.1. Common method bias test

Confirmatory factor analysis (CFA) was performed by Harman’s single-factor test and the possible presence of common method bias was examined. Factor analysis on the items of the basic psychological needs’ satisfaction, career decision self-efficacy, career outcome expectations, and career adaptability was conducted, and common factors were extracted (Zhou and Long, 2004). The results of the CFA indicated that the first factor explained 30.97% of the variance, which was less than the threshold of 40%. Therefore, the questionnaire used in this study was found to have no common method bias.

### 3.2. Descriptive statistical analysis

Descriptive statistical analysis was conducted for the results of the basic psychological needs satisfaction, career decision self-efficacy, career outcome expectations, and career adaptability (see Table 1). All four variables showed a significant positive correlation with each other.

**Table 1:** Results of descriptive statistical and correlation analysis.

Variables	M	SD	1	2	3	4
Basic psychological needs satisfaction	4.99	0.80	1			
Career decision self-efficacy	3.35	0.54	0.601^**^	1		
Career outcome expectations	2.98	0.31	0.549^**^	0.542^**^	1	
Career adaptability	3.42	0.50	0.556^**^	0.749^**^	0.428^**^	1

^*^p < 0.05, ^**^p < 0.01, and ^***^p < 0.001.

### 3.3. Mediating effect testing of career decision self-efficacy

Model 4 in PROCESS was used to test the mediating effect of career decision self-efficacy between basic psychological needs satisfaction and career adaptability with gender, grade, and major controlled. As shown in Table 2, basic psychological needs satisfaction significantly and positively predicted career decision self-efficacy and career adaptability. When career decision self-efficacy was simultaneously included in the prediction of career adaptability along with basic psychological needs satisfaction, both basic psychological needs satisfaction and career decision self-efficacy significantly and positively predicted career adaptability. Thus, Hypothesis 1 of the study was supported.

**Table 2:** Results of mediating effect testing of career decision self-efficacy.

Outcome variables	Predictor variables	*R*	*R* ^***2***^	*F*	*B*	Bootstrap 95% CI		*t*
						LLCI	ULCI	
Career adaptability		0.56	0.31	76.46^***^	0.35	0.31	0.39	9.98
	Gender				−0.07	−1.83	1.69	−0.07
	Grade				−0.01	−1.01	0.98	−0.03
	Major				−0.03	−0.98	0.92	−0.06
	BPNS				0.93	0.82	1.03	17.43^***^
Career decision self-efficacy		0.6	0.36	97.11^***^				
	Gender				−0.92	−3.86	2.03	−0.61
	Grade				0.41	−1.25	2.07	0.48
	Major				−0.05	−1.63	1.53	−0.06
	BPNS				1.75	0.36	0.44	19.73^***^
Career adaptability		0.76	0.58	187.95^***^				
	Gender				0.28	−1.1	1.65	0.39
	Grade				−0.17	−0.94	0.61	−0.42
	Major				−0.01	−0.75	0.73	−0.03
	BPNS				0.27	0.17	0.38	5.29^***^
CDSE					0.37	0.34	0.41	20.94^***^

BPNS, Basic psychological needs satisfaction; CDSE, Career decision self-efficacy. ^***^p < 0.001.

Furthermore, the Bootstrap procedure was employed with 5,000 resampling iterations to test the mediating effect of career decision self-efficacy between basic psychological needs satisfaction and career adaptability. As shown in Table 3, basic psychological needs satisfaction significantly predicted career adaptability, with a direct effect of 0.27 and a 95% confidence interval [0.17, 0.38]. The indirect effect mediated by career decision self-efficacy was 0.65, with a 95% CI [0.56, 0.76]. Both the CIs of the direct and indirect effects did not include zero. Therefore, career decision self-efficacy exhibited a significant mediating effect between basic psychological needs satisfaction and career adaptability, playing a partial mediating role. The mediating effect accounted for 69.62% of the total effect, supporting Hypothesis 2 of the study.

**Table 3:** Total effect, direct effect, and mediating effect.

	Effect	Boot SE	Bootstrap 95% CI		Relative effect size
			LLCI	ULCI	
Total effect	0.93	0.05	0.82	1.03	
Direct effect	0.27	0.05	0.17	0.38	30.38%
Mediating effect	0.65	0.05	0.56	0.76	69.62%

### 3.4. Moderated mediation model testing

Model 14 in PROCESS and the Bootstrap procedure were employed to test the mediating effect of career outcome expectations on the relationship between basic psychological needs satisfaction as the independent variable, career decision self-efficacy as the mediating variable, career outcome expectations as the moderating variable, and career adaptability as the dependent variable. As shown in Table 4, basic psychological needs satisfaction significantly and positively predicted career adaptability. Additionally, career decision self-efficacy demonstrated a significant positive prediction of career adaptability. However, career outcome expectations did not have a significant direct effect on career adaptability. Nevertheless, the interaction between career outcome expectations and career decision self-efficacy significantly and positively predicted career adaptability, with a significant moderating effect (*R*^2^ = 0.58, *F* = 135.58, *p* < 0.05). Hence, career decision self-efficacy was moderated by career outcome expectations in the pathway to career adaptability, impacting the latter half of the mediated effect. Hypothesis 3 of the study was supported.

**Table 4:** Moderated mediation model testing.

Variables	Coeff	SE	*t*	Bootstrap	95% CI
				LLCI	ULCI
Gender	0.38	0.7	0.55	−0.99	1.76
Grade	−0.23	0.4	−0.58	−1.01	0.55
Major	−0.03	0.38	−0.08	−0.77	0.71
Basic psychological needs satisfaction	0.28	0.05	5.17^***^	0.18	0.39
Career decision self-efficacy	0.38	0.02	20.11^***^	0.34	0.41
Career outcome expectations	−0.11	0.1	−1.09	−0.31	0.09
Career decision self-efficacy × career outcome expectations	0.01	0.00	2.30^***^	0.00	0.01
*R^2^*	0.58				
*F*	135.85				

^***^p < 0.001.

Career outcome expectations moderated the indirect effect of basic psychological needs satisfaction on college student’s career adaptability through career decision self-efficacy. As shown in Table 5, for college students with low levels of career outcome expectations, the indirect effect of basic psychological needs satisfaction on college student’s career adaptability through career decision self-efficacy was significant. Similarly, for college students with high levels of career outcome expectations, the indirect effect was also significant.

**Table 5:** Mediating effect at different levels of career outcome expectations.

Items	Career outcome expectations	Effect size	SE	Bootstrap 95% CI	
				LLCI	ULCI
Moderated mediating effect	M − 1SD	0.62	0.05	0.52	0.73
	M	0.66	0.05	0.56	0.76
	M + 1SD	0.70	0.06	0.60	0.81

**Figure 2:**
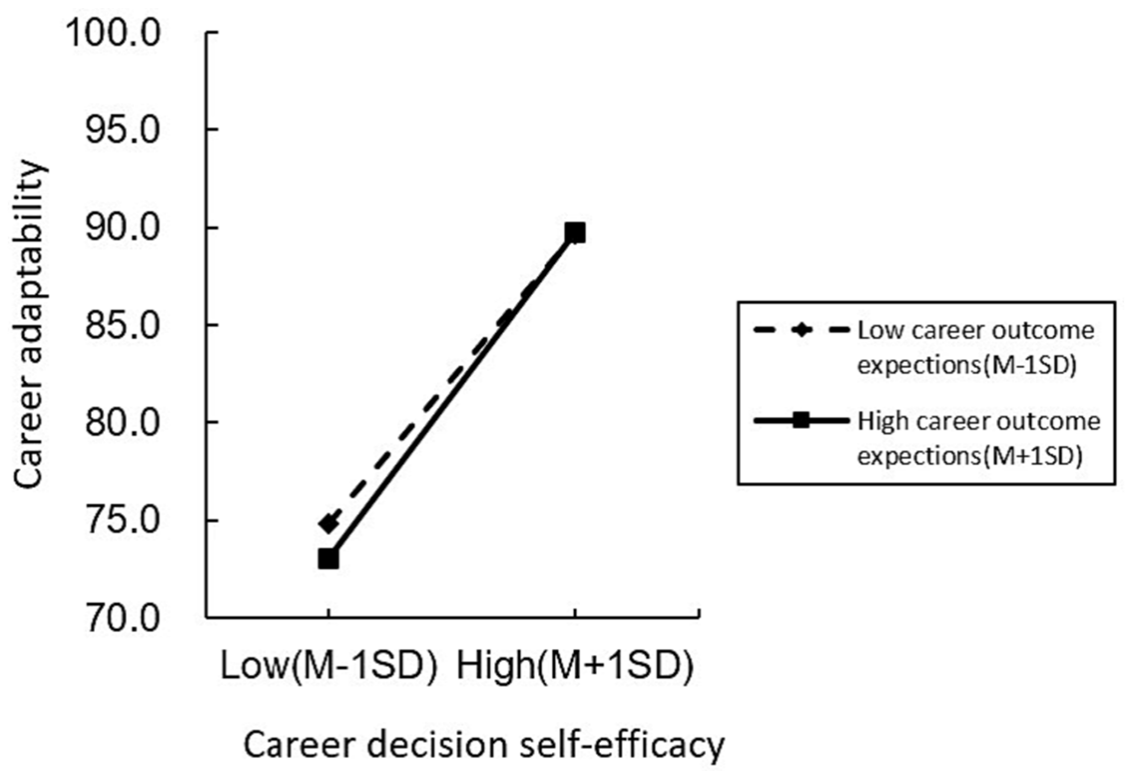
Mediating effect of career outcome expectations on the relationship between career decision self-efficacy and career adaptability.

In order to further test the moderating effect of career outcome expectations on career decision self-efficacy and career adaptability, simple slope analysis was carried out, and the career outcome expectations were grouped according to the mean plus or minus one standard deviation. Those above one standard deviation belonged to the high career outcome expectations group and the below belonged to the low career outcome expectations group. The results were shown in Figure 2. At the low level of career outcome expectations, the positive predictive effect of career decision self-efficacy on career adaptability was significant (*b*_simple_ = 0.36, SE = 0.02, *t* = 16.56, *p* < 0.001, 95% CI [0.31, 0.40]); at the high level of career outcome expectations, the positive predictive effect of career decision self-efficacy on career adaptability was also significant (*b*_simple_ = 0.4, SE = 0.02, *t* = 18.94, *p* < 0.001, 95% CI [0.36,0.44]). Therefore, as the level of career outcome expectations increased, the predictive effect of career decision self-efficacy on career adaptability would also increase.

## 4. Discussion

Currently, enhancing the career adaptability of college students is a practical challenge in addressing employment difficulties. This study is based on the basic psychological need theory and the social cognitive career theory. It aimed to investigate the relationship between college students’ basic psychological needs satisfaction and their career adaptability. Additionally, the study explored the role of career decision self-efficacy as a partial mediator in the relationship between basic psychological needs satisfaction and career adaptability. Moreover, the study examined the moderating effect of career outcome expectations on the impact of basic psychological needs satisfaction on career adaptability. Ultimately, the study aimed to establish the underlying mechanism of how basic psychological needs satisfaction influenced the career adaptability of college students.

The major results of this study lead to the following discussion points. First, the research found a significant positive correlation between the satisfaction of basic psychological needs and career adaptability in college students, which is consistent with other research results (Gulsen and Sahin, 2022). In addition, the basic psychological needs satisfaction can positively predict career adaptability. This meant that as the degree of basic psychological needs satisfaction in college students increased, so does the level of their career adaptability. According to the theory of basic psychological needs, the satisfaction of these needs acts as a central link between the external environment and individual motivation and behavior. When individuals’ autonomy, competence, and relatedness needs are fulfilled, it promotes the internalization of motivation, which in turn leads to the development of individual behaviors (Zhang et al., 2010). In other words, when college students have their autonomy, competence, and relatedness needs met in their daily lives, it not only promotes their mental well-being but also enhances their career adaptability. This increased career adaptability drives college students to focus more on their career development, stimulates curiosity about potential careers, and fosters career control and self-confidence. On the contrary, if the basic psychological needs are not met or are frustrated, it can have negative effects on their career development, particularly their career adaptability. This highlights the importance of basic psychological needs satisfaction as a significant predictor of career adaptability.

Second, the research found a significant positive correlation between the satisfaction of basic psychological needs and career decision self-efficacy, as well as a significant positive correlation between career decision self-efficacy and career adaptability. Career decision self-efficacy plays a partial mediating role in the relationship between basic psychological needs and career adaptability. In other words, the basic psychological needs satisfaction not only directly predicted career adaptability but also indirectly influenced it through career decision self-efficacy. This finding aligns with the principles of social cognitive career theory, which suggests that individual career-related behaviors are influenced by cognitive factors, particularly career decision self-efficacy (Lent et al., 1994). Studies have demonstrated that when college students’ three basic psychological needs are satisfied, it not only impacts their career focus, exploration, control, and self-confidence but also promotes their career-related decision. Additionally, when college students believe in their abilities in the career domain, they are more likely to engage in information collection, self-positioning, environmental positioning, and develop corresponding plans, which further enhances their career adaptability. Therefore, the basic psychological needs satisfaction has both a direct and indirect effect on the development of career adaptability, with the latter being mediated by career decision self-efficacy.

Further research found that career outcome expectations can regulate the direct impact of career decision self-efficacy on career adaptability. The study found that career outcome expectations significantly moderated the second half of the path from basic psychological needs satisfaction to career decision self-efficacy to career adaptability. Specifically, the interaction between career decision self-efficacy and career outcome expectations had a significant predictive effect on career adaptability. As the level of career outcome expectations increases, the impact of career decision self-efficacy on career adaptability becomes stronger. Career outcome expectations are crucial cognitive factors in that people’s behaviors are purposeful, and they tend to have certain expectations for the outcomes of their actions (Lent et al., 1994). These outcome expectations can enhance current behavioral motivations, such as material expectations (financial rewards), social expectations (recognition), and self-evaluation expectations (personal satisfaction) (Long et al., 2002). When college students have optimistic expectations regarding the outcomes they desire, it fuels their enthusiasm and active engagement in pursuing their goals. Simultaneously, they also enhance their belief in their abilities to achieve their desired outcomes, ultimately contributing to the realization of their goals. This means that career outcome expectations can strengthen the influence of career decision self-efficacy on college students’ career adaptability. Therefore, in the context of college students’ lives, it is crucial to not only provide adequate resources to fulfill their three basic psychological needs but also to assist them in enhancing their career decision self-efficacy and career outcome expectations. It will better support the development of their career adaptability.

In summary, this study constructed a moderated mediation model to validate the impact of college students’ basic psychological needs satisfaction on career adaptability and its underlying mechanisms. It has provided relevant research support and insights for enhancing college students’ development of career adaptability. The findings suggested that fulfilling college students’ three basic psychological needs and enhancing career decision self-efficacy and career outcome expectations are important pathways to promote the development of career adaptability.

## 5. Conclusion

The conclusions of this study are as follows: there is a significant positive correlation between college students’ basic psychological needs satisfaction, career decision self-efficacy, and career adaptability; college students’ basic psychological needs satisfaction can directly and positively predict career adaptability, and it can also indirectly influence career adaptability through career decision self-efficacy; career outcome expectations play a moderating role in the relationship between career decision self-efficacy and career adaptability.

There are some limitations in this study: in terms of research subjects, there are fewer participants in their third and fourth years of college, and subsequent research should increase the number of participants; The type of college student’s major is only a control variable in this study, and subsequent studies can be conducted to develop a study on the type of major; The study adopts a cross-sectional research design, which cannot determine the causality of variables, in future research, longitudinal study designs can be employed to improve the reliability of research results.

## Data availability statement

The raw data supporting the conclusions of this article will be made available by the authors, without undue reservation.

## Ethics statement

The studies involving humans were approved by Zaozhuang University, Zaozhuang. The studies were conducted in accordance with the local legislation and institutional requirements. The participants provided their written informed consent to participate in this study.

## Author contributions

MX: Writing – original draft. HL: Writing – review & editing. QG: Writing – review & editing.
